# Genome wide association study of frost tolerance in wheat

**DOI:** 10.1038/s41598-022-08706-y

**Published:** 2022-03-28

**Authors:** Behnaz Soleimani, Heike Lehnert, Steve Babben, Jens Keilwagen, Michael Koch, Fernando Alberto Arana-Ceballos, Yuriy Chesnokov, Tatyana Pshenichnikova, Jörg Schondelmaier, Frank Ordon, Andreas Börner, Dragan Perovic

**Affiliations:** 1 Federal Research Centre for Cultivated Plants, Institute for Resistance Research and Stress Tolerance, Julius Kühn-Institut (JKI), Erwin-Baur-Str. 27, 06484 Quedlinburg, Germany; 2 Federal Research Centre for Cultivated Plants, Institute for Biosafety in Plant Biotechnology, Julius Kühn-Institut (JKI), Erwin-Baur-Str. 27, 06484 Quedlinburg, Germany; 3 Institute of Agricultural and Nutritional Sciences, Martin Luther University Halle-Wittenberg (MLU), Betty-Heimann-Str. 5Saxony-Anhalt, 06120 Halle (Saale), Germany; 4 Deutsche Saatveredelung AG (DSV), Weißenburger Str. 5, 59557 Lippstadt, Nordrhein-Westfalen, Germany; 5 Leibniz Institute of Plant Genetics and Crop Plant Research (IPK), Resources Genetics and Reproduction, Correnstraße 3, 06466 Seeland OT Gatersleben, Germany; 6 Agrophysical Research Institute (AFI), Grazhdanskii prosp. 14, 195220 St. Petersburg, Russia; 7 Institute of Cytology and Genetics of Siberian Branch of the Russian Academy of Sciences, Prospekt Lavrentyeva, 10 630090 Novosibirsk, Russia; 8 Saaten-Union Biotec GmbH, Hovedisser Str. 94, 33818 Leopoldshoehe, Nordrhein-Westfalen, Germany

**Keywords:** Biotechnology, Genetics, Plant sciences

## Abstract

Winter wheat growing areas in the Northern hemisphere are regularly exposed to heavy frost. Due to the negative impact on yield, the identification of genetic factors controlling frost tolerance (FroT) and development of tools for breeding is of prime importance. Here, we detected QTL associated with FroT by genome wide association studies (GWAS) using a diverse panel of 276 winter wheat genotypes that was phenotyped at five locations in Germany and Russia in three years. The panel was genotyped using the 90 K iSelect array and SNPs in FroT candidate genes. In total, 17,566 SNPs were used for GWAS resulting in the identification of 53 markers significantly associated (LOD ≥ 4) to FroT, corresponding to 23 QTL regions located on 11 chromosomes (1A, 1B, 2A, 2B, 2D, 3A, 3D, 4A, 5A, 5B and 7D). The strongest QTL effect confirmed the importance of chromosome 5A for FroT. In addition, to our best knowledge, eight FroT QTLs were discovered for the first time in this study comprising one QTL on chromosomes 3A, 3D, 4A, 7D and two on chromosomes 1B and 2D. Identification of novel FroT candidate genes will help to better understand the FroT mechanism in wheat and to develop more effective combating strategies.

## Introduction

Bread wheat (*Triticum aestivum* L.) is an allohexaploid (2n = 6x = 42, AABBDD) species derived from two hybridization events in the region of the Near Eastern Fertile Crescent^1^. The spread of domesticated hexaploid wheat from the Near Eastern Fertile Crescent to today’s growing regions required phenological adaption to different environments, e.g., selection of spring/winter types or phenotypes with reduced photoperiod sensitivity^2, 3^. In general, three different types of bread wheat adapted to specific environments according to the vernalization requirement, which is necessary for the transition from the vegetative to the generative stage, are known (spring, facultative and winter type )^4^. The exposure to cold temperature needed for the vernalization response varies between 2–4 weeks and 4–6 weeks in semi and strong winter types, respectively^5^. Winter wheat types are higher yielding compared to spring wheat types, because they can take advantages of autumn rainfall^6^ and have a longer vegetation period^7^. Today, wheat is cultivated on around 220 M ha worldwide, which resulted in an annual production of 757 M tones in 2018^8^. It is estimated that approximately one-third of the wheat growing area is cultivated with winter or facultative wheat types. Winter hardy wheat varieties are mostly needed in the Great Plains of North America, the Russian Federation, as well as Eastern Turkey, Northwestern Iran and China^9^.

Low temperatures significantly reduce yield performance in Australia^10^, Europe^11^ and United States^12^. Frost damage is observed when sensitive tissue of plants is faced with low temperature in different growth stages^13^. Economic damage of frost events on crop performance depends on the time point of occurrence^14^. Due to the cold acclimation phenomenon, winter cereals survive frost by regulating their metabolism at low temperatures and protecting critical structures of cells against freezing temperatures^15, 16^.

In order to reduce the negative effects of frost on crop production, it is necessary to identify genes or genomic regions involved in FroT^17^. The mechanism of plants that describes the response to low temperature by increasing the freezing tolerance is called cold acclimation. Some physiological and biochemical changes occur during cold acclimation, e.g., soluble sugars, proline and cold-resistance proteins are synthesized to protect proteins at the physiological level^18^. These substances play a role in cold stress response of plants through the regulation of the osmotic potential, ice crystal formation, stability of cell membranes and reactive oxygen species. Several components encompassing messenger molecules, protein kinases, phosphatases and transcription factors assumed to be involved in cold-stress signaling pathways have been reported during the last decades^19^.

Two important FroT loci namely FROST RESISTANCE 1 (FR-A1) and FROST RESISTANCE 2 (FR-A2) are located on the long arm of wheat chromosome 5A. These loci influence freezing tolerance and winter hardiness. The first locus is closely located to the *VRN-A1* gene, but no information on the effect of this gene on the FR1 locus is known. *Vrn-B1* and *Vrn-D1* genes are mapped on the long arm of chromosome 5B and 5D, respectively. *VRN-A1* has a major effect in the determination of spring/winter habit^20^ and plays a major role in FroT^15^. Regarding other genes involved in FroT, the *ICE (inducer of CBF expression*)—*CBF* (*C-repeat binding factor*)—COR (cold-responsive or cold-regulated) pathway has been known as the main cold signaling pathway in many plant species^21–23^. Under low temperatures, DELLA releases ICE1 from its JASMONATE ZIM-DOMAIN (JAZs) enabling the induction of CBF genes, which are members of the AP2/ERF (APETALA2/ETHLENE RESPONSIVE FACTOR) family^24^. CBF genes bind to C-repeat/ dehydration-responsive elements (CRT/DRE) and regulate the expression of cold-responsive/late embryogenesis-abundant (COR/LEA) genes^25^.

In addition to the ICE–CBF–COR pathway, vernalization genes e.g., VRN1, VRN2 and VRN3 respond to low temperature through the flowering pathway. Therefore, changes in the regulatory regions of vernalization genes cause a delay in flowering time in plants^26^. For instance, VRN1 reduces FroT by decreasing the transcript level of CBF and COR genes^27^. Therefore, a delay in flowering time increases FroT, which indicates a connection between the flowering and cold response pathway by the interaction of VRN1 with CBF and COR genes.

Genome wide association studies (GWAS) are widely applied in many crop plants to identify quantitative trait loci (QTLs) associated with traits of interest^28–31^. Development of high-throughput single nucleotide polymorphism (SNP) genotyping platforms, e.g., Illumina^32^ and Affymetrix^33^ enabled the conduction of GWAS in plants and became a useful approach to detect QTL and allelic variation for complex traits^34, 35^. GWAS was successfully applied in wheat to identify QTL regions associated with abiotic stress tolerance (e.g.^36^), yield components (e.g. [37, 387]), grain quality (e.g.^39^) or diseases resistance (e.g.^40^). However, up to now, only a few studies were published dealing with FroT candidate identification by GWAS^41^.Nevertheless, during the last decade, several FroT QTL mapping analyses using bi-parental populations were conducted and QTL on different wheat chromosomes (with the exception of chromosome 4D) were identified^42–46^. However, the majority of these QTL regions was identified by bi-parental QTL studies. Vagujfalvi et al.^15^ pointed out that ten wheat chromosomes were assumed to be involved in the regulatory gene networks associated with FroT. However, until now, the majority of genes which are assumed to be involved in FroT have been identified on chromosomes 5A, 5B and 5D^20, 25, 47^.

Therefore, the aims of the present study were (i) to conduct GWAS to identify genome regions associated with FroT, (ii) to investigate potential candidate genes from QTL regions using the wheat reference genome and (iii) to compare our results with previously published FroT regions and genes in winter wheat.

## Material and methods

### Phenotypic data

A panel of 276 bread wheat genotypes from 31 countries was evaluated for FroT. This panel comprised 83, 4, 143 and 46 genotypes from Asia, Australia, Europe and USA, respectively (Supplementary Table S1). Out of these 216, 48, and 12 were cultivars, breeding lines and doubled haploid lines, respectively. In a previous study, Babben et al.^25^ used 235 out of the 276 genotypes under investigation to identify polymorphism in known FroT genes and to conduct a candidate gene based association study (CGAS).

All genotypes were tested in four environments during 2012 and 2013 (Gatersleben, Germany; Ranzin, Germany; Puskin, Russia; Roshchinskiy, Russia) and one environment in 2012 and 2014 (Novosibirsk, Russia), according to Babben et al.^25^. Genotypes were tested in a random design in double rows and two replications per genotype. However, in Roshchinskiy, genotypes were tested as a miniplot (2.5m^2^) trial with only one replication. FroT was assessed as percentage winter survival of plants per plot for each genotype after winter (0% = all plants died, 100% = no plant died; for further information see Babben et al.^25^). The quality check of phenotypic data was done as described by Babben et al.^25^. Least Square means (LSmeans) per genotype were estimated by fitting a mixed linear model in SAS 9.4^48^ (for further information please see Babben et al.^25^).

### Genome wide association studies (GWAS)

Genotyping of the 276 wheat genotypes was conducted at Trait Genetics, Gatersleben (Germany), by using the 90 K iSelect array (Illumina Inc., San Diego, USA). Flanking sequences of the 90 K array were mapped against the reference genome of Chinese Spring RefSeqv1.0^49^. All mapped markers with more than 30% missing values were excluded. The resulted marker set was imputed by using the Beagle 4.1 software^50^. Next, the imputed marker data set was filtered for minor allele frequency (MAF) ≥ 3% and heterozygosity ≤ 12.5%. The filtered marker data set was combined with 182 SNP markers indicating polymorphisms in 15 candidate genes for FroT known from a previously published study^25^. This final marker data set consisting of 17,566 SNP markers was used for LD decay and GWAS.

To estimate linkage disequilibrium (LD) and to determine LD decay the software package R was used (R Core Team. 2014, packages “genetics” and “LDheatmap”^51, 52^). The LD was estimated as squared allelic correlation (r^2^) between all pairs of markers within a chromosome. For graphical display, the genetic distances between markers in base pairs were plotted against the estimated r^2^. The critical value of r^2^ was set to r^2^ = 0.2 as described by Voss-Fels et al.^53^. Furthermore, a smooth locally weighted polynomial regression (LOESS) curve was fitted to calculate the LD decay . Finally, the LD decay was determined as intersection point of the LOESS curve and the critical r^2^ value^54^. LD decay was estimated for each chromosome and across all 21 wheat chromosomes.

In order to get comparable results with previously published analysis on the same material, a reduced marker set (249 markers, for further information see^25^) was used to determine population structure and to calculate kinship matrix.

Kinship matrix was calculated based on Roger’s distance for each pairwise genotype – genotype combination^55^. Population structure was investigated by using Bayesian cluster analysis implemented in Structure^56^ and principal coordinates analysis (PCoA) implemented in the DARwin 6 software^57^. To determine the population structure by using the software package STRUCTURE (source), ten independent runs were performed setting the number of populations (k) from 1 to 10. Furthermore, the number of burn-in and Markov Chain Monte Carlo (MCMC) iterations was set to 100,000. To determine the optimal number of subpopulations, the Evanno method (ΔK method) implemented in the software package STRUCTUTRE HARVESTER version 2.3.4^58^ was used.

GWAS was conducted by using the software package TASSEL 5^59^. A compressed Mixed Linear Model (CMLM) was used to examine associations between SNP markers and FroT data. Two association models were tested: 1) Q + K model (CMLM with Q-matrix and K-matrix as correction for population structure and kinship relationship); 2) K model (CMLM with K-matrix as correction for kinship relationship). All marker trait associations with LOD ≥ 4 (-log_10_ of *P* value) were assumed to be significantly associated with FroT according to Babben et al.^25^ and Zhao et al.^41^. All markers, which were significantly associated with FroT, were assigned to QTL regions according to their chromosomal position and the estimated LD decay (3.5 million base pairs). The peak marker of each QTL region is defined by the highest LOD value. Markers within a distance of ± 3.5 million base pairs to the QTL peak marker were assigned to one QTL region. Genes located within the QTL regions were identified based on their position on the reference genome of Chinese Spring^49^. All high and low confidential (HC and LC) genes located within a QTL region were identified. Additionally, published functional gene annotations^49^ were used to identify gene onthology (GO) terms associated with FroT. All GO terms associated with frost or cold tolerance were downloaded from the QuickGO website (https://www.ebi.ac.uk/QuickGO/). Genes within QTL regions were filtered based on GO terms associated with frost or cold tolerance (GO, Supplementary Table S2).

### Identification of candidate genes via BLASTn

The sequences of associated candidate genes to FroT were used to identify gene IDs. For this purpose, a BLASTn analysis (nucleotide Basic Local Alignment Search Tool) from National Center of Biotechnology Information (NCBI; https://www.ncbi.nlm.nih.gov/) based on the whole coding sequence (CDS) of candidate genes was used. These sequences were aligned to *Triticum* species with default settings. Perfect match or high similarity was identified based on 100% query coverage, an Expect (E) value of 0 and an identity higher than 99%.

## Results

Genotyping of the 276 wheat accessions resulted in a raw data set of 81,587 SNP markers. In total, 54,340 markers were excluded from further analyses, due to the absence of a hit or no unique map position according to the reference genome sequence of Chinese Spring^49^. The 27,247 uniquely mapped markers were filtered for minor allele frequency (≥ 3%) and heterozygosity (≤ 12.5%), resulting in a set of 17,566 (17,384 90 K SNPs and 182 CG polymorphic sites) informative markers.

The LD decay ranged between 609,686 bp (chromosome 6B) and 8,434,298 bp (chromosome 1D). However, it was not possible to estimate LD decay for chromosome 4D. The LD decay across all chromosomes was 3,377,883 bp (Supplementary Table S3). The calculated LD decay was used to define QTL regions.

Population structure was determined by a Bayesian cluster analysis implemented in in the software package Structure and a PCoA implemented in DARwin 6. The Bayesian cluster analysis revealed an optimal number of K = 2 or K = 3 subpopulations. Due to the origin of the genotypes (North America, Asia and Europe), K = 3 was used as optimal number of subpopulations (Figure S1). Genotypes were assigned to one of the three subpopulations based on their membership coefficients. However, genotypes with membership coefficients < 0.7 to a subpopulation were considered as admixture^60^. In total, 23, 78, 88 and 87 genotypes were assigned to subpopulation 1, 2, 3 or the admixed group. Additionally, a PCoA plot was used to visualize the results of the Bayesian cluster analysis. The first and the second PCoA explained 8.4% and 4.7% of the whole variance. Results of both analyses pointed to a weak to moderate population structure (Figure S2).

The compressed mixed linear model with correction for kinship relatedness (CMLM with K) turned out to be the most appropriate GWAS model (Figure S3). Therefore, GWAS was conducted based on the CMLM K model.

**Figure 1:**
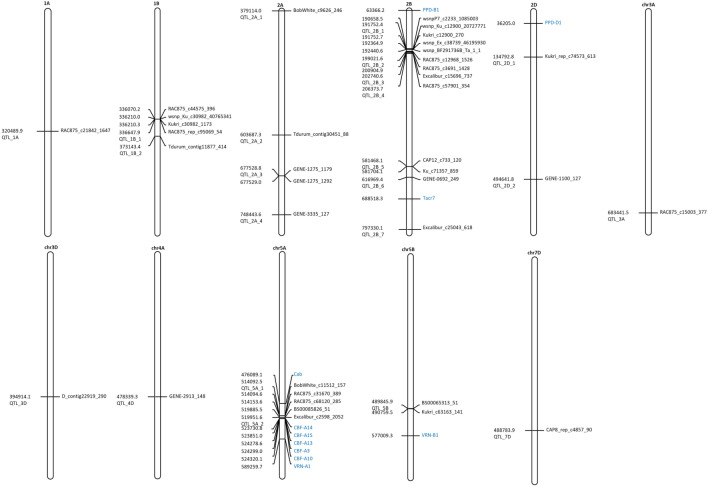
Genetic map of identified QTLs for FroT on wheat chromosomes: the identified markers are shown on the different chromosomes. Eleven candidate genes analysed by Babben et al.^25^ are assigned with blue color.

In total, 53 markers were found to be significantly associated with FroT at a significance threshold of LOD ≥ 4 (Supplementary Table S4). Out of these, 16 SNPs were associated with polymorphisms in a candidate gene for FroT (*CBF-14* on chromosome 5A) previously published by Babben et al.^25^ (Supplementary Table S4). The significantly associated markers were assigned to 23 QTL regions, which explained with a range of 6.1% to 16.2% from the total phenotypic variance. These QTLs were located on 11 chromosomes (1A, 1B, 2A, 2B, 2D, 3A, 3D, 4A, 5A, 5B and 7D) (Table 1, Fig. 1 and Supplementary Table S4), whereby the majority of significantly associated markers was detected on chromosome 2B (13 markers, Supplementary Table S4 and Fig. 1).

**Table 1:** QTL regions associated with FroT in wheat (LOD ≥ 4).

QTL name	Chromosome	Start^**a**^	End^**a**^	Peak marker ^**b**^	Pos^**c**^	P value	LOD^**d**^	Major allele^**e**^	Effect of the allele	QTL reported in previous studies	Identified genes in QTL region in *Triticum* species
QTL_1A	chr1A	316989869	323989869	RAC875_c21842_1647	320489869	2.13E-05	4.7	A	33.0	^61^	
QTL_1B_1	chr1B	333147904	340147904	RAC875_rep_c95069_54	336647904	2.06E-06	5.7	A	11.6	Novel	
QTL_1B_2	chr1B	369643402	376643402	Tdurum_contig11877_414	373143402	5.29E-05	4.3	A	11.9	Novel	bHLH, MYC2-like and Dehydration responsive element binding transcription factor
QTL_2A_1	chr2A	375613997	382613997	BobWhite_c9626_246	379113997	2.57E-05	4.6	A	30.0	^42, 62^	Low temperature and salt responsive protein
QTL_2A_2	chr2A	600187297	607187297	Tdurum_contig30451_88	603687297	1.13E-09	8.9	A	55.4	^42, 62^	cold shock domain protein 1
QTL_2A_3	chr2A	674028807	681028807	GENE-1275_1179	677528807	7.89E-06	5.1	A	17.3	^42, 62^	two WCOR15, bHLH and LEA 19-like
QTL_2A_4	chr2A	744943578	751943578	GENE-3335_127	748443578	4.97E-05	4.3	A	28.2	^42, 62^	bHLH, bHLH112-like and three FTs (putative)
QTL_2B_1	chr2B	188940604	195940604	wsnp_Ku_c12900_20727771	191752438	4.55E-07	6.3	A	28.8	^42, 61^	MYB8
QTL_2B_2	chr2B	195521553	202521553	RAC875_c12968_1526	199021553	6.24E-06	5.2	A	17.9	^42, 61^	RSL4, bHLH
QTL_2B_3	chr2B	199240571	206240571	Excalibur_c15696_737	202740571	6.24E-06	5.2	A	17.9	^42, 61^	
QTL_2B_4	chr2B	202873684	209873684	RAC875_c57901_354	206373684	6.24E-06	5.2	A	17.9	^42, 61^	
QTL_2B_5	chr2B	577968073	584968073	CAP12_c733_120	581468073	5.69E-05	4.2	A	17.0	^42, 61^	bHLH, anthocyanin regulatory R-S protein-like
QTL_2B_6	chr2B	613469385	620469385	GENE-0692_249	616969385	7.89E-06	5.1	A	17.3	^42, 61^	
QTL_2B_7	chr2B	793830074	800830074	Excalibur_c25043_618	797330074	1.33E-05	4.9	A	8.1	^42, 61^	
QTL_2D_1	chr2D	131292774	138292774	Kukri_rep_c74573_613	134792774	1.92E-05	4.7	A	-7.1	Novel	
QTL_2D_2	chr2D	491141738	498141738	GENE-1100_127	494641738	5.69E-05	4.2	A	17.0	Novel	
QTL_3A	chr3A	679941459	686941459	RAC875_c15003_377	683441459	2.66E-05	4.6	A	9.1	Novel	ICE1, bHLH and bHLH35-like
QTL_3D	chr3D	391414092	398414092	D_contig22919_290	394914092	3.72E-05	4.4	A	10.1	Novel	
QTL_4A	chr4A	474839274	481839274	GENE-2913_148	478339274	7.47E-05	4.1	A	31.1	Novel	
QTL_5A_1	chr5A	510592445	517592445	BobWhite_c11512_157	514092445	4.69E-05	4.3	A	18.4	^45, 46^	bHLH, MYC2-like, bHLH14-like three FPAs and REM18
QTL_5A_2	chr5A	516451564	523451564	Excalibur_c2598_2052	519951564	1.65E-05	4.8	A	18.5	^41, 44^	bHLH, two MYC2-like, PHYA, CBFIIId-17.1 (CBF-A17), CBFIVd-9.1 (CBF-A9), CBFIVd-26.2 (CBF-A26), CBFIVa-25.3 (CBF-A25), CBFIVd-A22 (CBF-A22), CBFIVa-A2 (CBF-A2), CBFIVd-4.1 (CBF-A4), CBFIIID-A19 (CBF-A19), CBFIIId-24.2 (CBF-A24), CBF-A14, CBF-A15, CBFIIId-16.1 (CBF-A16) and three CBFs
QTL_5B	chr5B	486345892	493345892	BS00065313_51	489845892	4.69E-05	4.3	A	18.4	^41, 45^	bHLH, two bHLH14-like, two MYC2-like, CBFIVd-B9 (CBF-B9), CBFIVd-B22b (CBF-B22), CBFIVd-B4 (CBF-B4), CBFIVb-B20 (CBF-B20) and CBFIVb-21.1 (CBF-B21) and five CBFs
QTL_7D	chr7D	485283857	492283857	CAP8_rep_c4857_90	488783857	9.94E-05	4.0	A	35.9	Novel	

^a^Start and End position of the QTL interval.

^b^Name of the Peak marker.

^c^Position of the Peak marker.

^d^-Log10 of p value (LOD).

^e^Major allele that influence FroT.

All identified QTL regions were screened for known functional genes located within the QTL regions. In total, 3,112 HC and 2,004 LC genes were identified within the QTL regions (Supplementary Table S2). The number of low confidence (LC) and high confidence (HC) genes per interval ranged between 111 and 589. As mentioned above, 16 of the markers significantly associated with FroT indicate polymorphisms in the *CBF-A14* gene located within the QTL region QTL_5A_2 on chromosome 5A. The *CBF 14* gene is known as a putative candidate gene for FroT in wheat^25^ and in barley^63^. Additionally, all LC and HC genes located within the QTL regions were screened for GO terms associated with cold stress or FroT. In total, in 23 QTLs from the present study a set of 5116 genes was annotated according to the Chinese Spring reference genome (Supplementary Table S2). Nevertheless, at 11 out of 23 QTLs (on chromosomes 1B, 2A, 2B, 3A, 5A and 5B) 53 candidate genes (CGs) related to FroT were identified. Out of 53 FroT related CGs, 43 are high confidence and 10 are low confidence (Supplementary Table S5).

At chromosome 2A four QTLs containing 1317 Chinese Spring annotated genes, of which two HC and three LC are related to FroT, were identified. Interestingly, two of these genes code for a *cold-responsive protein* (*WCOR15,* QTL_2A_3) and the other one was a *cold shock domain protein 1* (QTL_2A_2). In addition, a gene coding for a low temperature and salt responsive protein was identified within QTL_2A_1 on chromosome 2A. Furthermore, genes coding for 16 *basic helix-loop-helix* (*bHLH*) transcription factors were detected on chromosome 1B, 2A, 2B, 3A, 5A and 5B. Three genes coding for *flowering locus T-like proteins* (*FT-like*) and four genes for *flowering time control proteins* (*FPA*) were identified on chromosome 2A and 5A, respectively. An important gene involved in the FroT pathway, *ICE1*, was observed on chromosome 3A. In addition, 25 *CBF* genes have been identified. Fifteen of them were located on chromosome 5A (specifically on the locus of frost resistance A2 (FR-A2)) and 10 on chromosome 5B. Seventeen specific gene names out of these 25 *CBF* candidate sequences were identified via BLASTn (Supplementary Table S6). Twelve and five out of these 17 identified genes were located at the FR-A2 locus on chromosome 5A and 5B, respectively.

Based on the obtained BLASTn results, 13 out of 16 transcription factor candidate sequences were identified on five different wheat chromosomes (Supplementary Table S6). Out of six identified *MYC2*-like transcription factors, one, three and two were located on chromosome 1B, 5A and 5B, respectively. The five *bHLH* transcription factors comprised three *bHLH14-like* transcription factors (5A and 5B), one *bHLH35-like* (3A) and one *bHLH122-like* (2A), respectively. The genes *ROOT HAIR DEFECTIVE6‐LIKE 4* (*RSL4*) and *anthocyanin regulatory R-S protein-like* were identified on chromosome 2B.

Additionally, we identified further possible candidate genes like a dehydration responsive element binding transcription factor (1B), a *late embryogenesis abundant* (*LEA*) *19-like protein* (2A), a *MYB8* transcription factor (2B), a *AP2/B3-like transcriptional factor protein* (*REM18*, 5A) and *Phytochrome A* (*PHYA*, 5A) (Supplementary Table S6).

## Discussion

Bread wheat is grown worldwide in temperate latitudes and subtropical regions^64^ and constitutes the main source of proteins and calories in human diets^65^. However, many wheat-growing areas are regularly exposed to heavy low temperature events during the early stage of wheat development^9^ causing severe yield losses^66^. Therefore, FroT is an important trait in breeding in order to improve winter hardiness of wheat^16^.

In the recent years, high-throughput sequencing technologies fostered the availability of large SNP data sets and therefore the conduction of population genetic and GWAS studies^32^. Furthermore, these technologies made the first fully annotated reference genome sequence of wheat available^49^. Altogether, this progress in plant genetics and genomics helps to increase the understanding of wheat biology and the molecular basis of important agronomic traits^67, 68^. Based on this progress, this study aimed to identify QTL regions and candidate genes associated with FroT in wheat.

Recently, QTL mapping studies or GWAS identified several QTL regions associated with FroT in wheat on all wheat chromosomes except chromosome 4D^25, 41, 43–46, 69, 70^. In general, it is difficult to compare QTL regions identified by different studies using different marker systems and different genetic maps. Therefore, to compare the records from literature with findings of this study, known flanking sequences of markers associated with QTL for FroT in literature were mapped to the reference genome sequence of Chinese Spring^49^. However, flanking markers were not available for all published QTL regions. Hence, these QTLs could not be anchored on the reference genome sequence of Chinese Spring. Furthermore, for some flanking marker sequences no unique position on the reference genome sequence of Chinese Spring^49^ could be identified. Therefore, for markers and QTL regions that could be not uniquely mapped to the reference genome, comparison with the results of this study was conducted based on the chromosome (Supplementary Table S7).

To get comparable results, identified candidate genes in the current study with previous study^25^, LOD ≥ 4 was subjected as threshold for significantly associated markers with FroT. In addition, Zhao et al.^41^ reported associated significant markers with FroT with LOD ≥ 4. The *P* value was also adjusted by Bonferroni-Holm (LOD ≥ 5.5) correction in the present study. Finally, since only three markers were identified at Bonferroni-Holm threshold at LOD ≥ 5.5, we considered markers with LOD ≥ 4 for further analysis. In total, 53 markers were found to be significantly associated (LOD ≥ 4) with FroT in this study. These markers were assigned to 23 QTL regions on 11 chromosomes (1A, 1B, 2A, 2B, 2D, 3A, 3D, 4A, 5A, 5B and 7D).

In the present study, three out of 23 identified QTL regions namely QTL_5A_1, QTL_5A_2 and QTL_5B are overlapping with previously reported QTL regions^41, 44–46^. It is known that important genes associated with FroT are located on chromosome 5A, e.g., *CBF* genes and *VRN* genes. In this study, 17 *CBF* genes, three *bHLH* family transcription factors and one *FPA* gene were identified within QTL regions associated with FroT on chromosome 5A and 5B. Polymorphisms within two out of 17 identified *CBF* genes (*CBF-A14* and *CBF-A15*) were previously detected by CGAS^25^. In total, 16 markers associated with polymorphic sites for *CBF-A14* were significantly associated with FroT in the present study.

In plants, Hormones and photoreceptors, such as phytochromes, regulate the expression of the CBF regulon as a response to the modulated spectrum of the incident light, to enhance cold acclimation and freezing tolerance in plants^71^. The positive role of *PHYA* on the transcription levels of *CBF* pathway genes is reported in tomato^72^, wheat and barley^73, 74^. In the present study, *PHYA* was associated with a QTL for FroT on chromosome 5A.

Furthermore, twelve QTL regions, i.e., QTL_1A, QTL_2A-1, QTL_2A-2, QTL_2A-3, QTL_2A-4, QTL_2B_1, QTL_2B_2, QTL_2B_3, QTL_2B_4, QTL_2B_5, QTL_2B_6 and QTL_2B_7 are potentially overlapping with previously reported QTLs on the same chromosomes^42, 61, 62^. However, due to the unavailability of flanking markers or due to the fact that the flanking sequences of available markers could not be anchored on the reference genome^49^; it was not possible to confirm this assumption. Three candidate genes associated with cold or low temperature tolerance were identified on chromosome 2A. The QTL_2A_1, QTL_2A_2 and QTL_2A_3 were co-localized with genes coding for *Low temperature and salt responsive protein*, *cold shock domain protein 1*, *WCOR15* and *LEA 19-like protein*, respectively.

CORs are referred to as proteins encoded by cold-responsive or cold-regulated genes, which are involved in the cold tolerance acquisition and subsequent freezing tolerance. These genes, i.e., LEA, stress responsive protein (SRP), cold induced (KIN) and low temperature induced (LTI)^21^, increase cold tolerance in plants. For instance, accumulation of COR/LEA proteins during cold acclimation protects cell structures and functions from freezing damage^62^. The *Wcor15* is expressed under low temperature^75^ and encodes a chloroplast-targeted protein in wheat and barley.

QTL_2A_4 includes a gene coding for Flowering Locus T-like protein. It has been shown that flowering time genes are not only responsible for the transition from the vegetative to the reproductive phase, but also involved in various environmental stress responses. The relation between flowering and cold response is well known^76^.

For eight of the QTLs associated with FroT in this study, we did not find any evidence that these QTL were previously reported in literature. These QTLs are located on chromosome 1B, 2D, 3A, 3D, 4D and 7D and will be discussed in the following.

One out of two identified QTL regions on chromosome 1B contains a gene encoding a *bHLH* transcription factor. *bHLH* transcription factors play diverse roles in different physiological processes^77^. Several studies have been shown that *bHLH* is involved in different responses, which are provoked by cold and other abiotic stresses in Arabidopsis and rice^78–82^. Wang et al.^83^ identified 159 *bHLH* -encoding genes in wheat, which are involved in abiotic and biotic stress response. Furthermore, they pointed out that 98.7% of these genes are associated with more than one stress. In addition, the expression of these genes under different stresses was evaluated. In total, 38.44% of these genes were upregulated under cold stress in wheat^83^.

The identified *MYC2-like transcription factor* is also called *JAM* (*JASMONATE ASSOCIATED MYC2-LIKE*) and *bHLH14* is called *JAM1*. Both transcription factors are members of the *IIId bHLH* subfamily, which is phylogenetically closely related to *MYC* proteins interacting with *JAZ* proteins. These *bHLH* subfamily acts as transcription repressor of *MYC2* and so as a negative regulator of jasmonate mediated response^84, 85^. Furthermore, Xiang et al.^86^ described the role of cold induced transcription factor *bHLH112*, which promotes a positive regulation of *AP2/ERF* transcription factor in *Artemisia annua* and Jiang et al.^87^ identified that *bHLH35* is involved in cold tolerance in *Anthurium andraeanum*.

In addition, we identified one QTL region on chromosome 3A. This region comprises transcription factor ICE1 and *bHLH* transcription factor genes. The transcription factor *ICE1* is known as an important gene involved in freezing tolerance (*ICE* -*CBF* -*COR*) pathway. *ICE1* genes are known in wheat, but until now, no *ICE1* gene was found to be located within a QTL region associated with FroT. Two *ICE* homologs, i.e., *TaICE41* (accession no. EU562183) and *TaICE87* (accession no. EU562184) have been identified in wheat^21^. The identified AP2/B3-like transcriptional factor protein (REM18) on chromosome 5A is also a member of the DREB/ERF subfamily and it is accordingly maybe involved in FroT^88, 89^.

The *bHLH35-like* gene was identified on chromosome 3A. Less knowledge is available for this gene. Jiang et al.^87^ have reported the positive role of *bHLH35* in response to abiotic stresses in Arabidopsis. They reported that *bHLH35* from *Anthurium andraeanum* (*AabHLH35*) increases stress tolerance to cold and drought in Arabidopsis. The expression of *CBF1* and *COR15A* in wild type (WT) and *AabHLH35* transgenic lines of Arabidopsis was significantly increased under cold stress compared to control plants. Expression of *COR15A* was threefold higher in *AabHLH35* transgenic lines relative to WT lines. Therefore, they assumed that *AabHLH35* might promote *COR15A* expression in response to cold stress. Furthermore, *OsbHLH35* increased salinity tolerance in rice^90^ and *PebHLH35* from *Populus euphratica* increases drought tolerance in Arabidopsis^91^.

**Figure 2:**
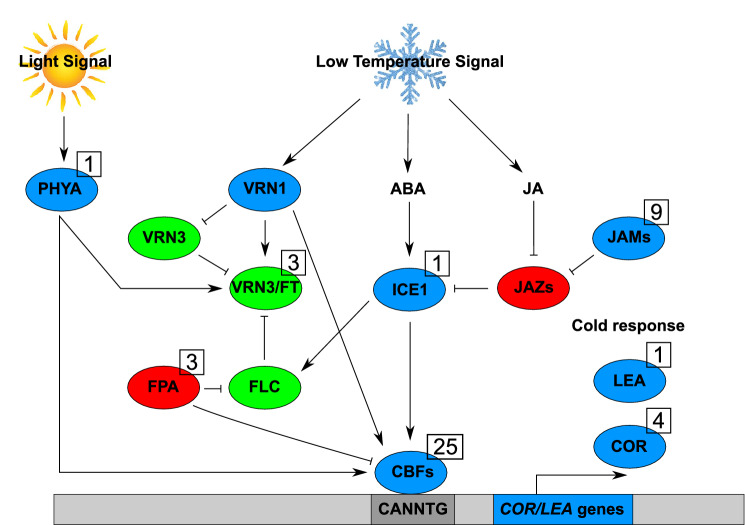
Cold response regulatory pathway based on identified genes for FroT in the current study: The major key transcription factor ICE1 becomes initiated via low temperature signals and ABA, repressed via JAZ proteins and control transcription of *CBF* genes. *CBFs* regulate the expression FroT response genes (*COR/LEA* genes). On the other site vernalization genes regulate flowering and specially *VRN1* is involved in FroT response. FPA promotes flowering and represses FroT. PHYA which are regulated via light signals promotes flowering and also FroT. Ovals represents proteins and the bar on the bottom genomic DNA. The blue color indicated positive FroT regulators, red repressors and green flowering proteins. The gray box (CANNTG) in the bar on the bottom stays for CBF promotor interaction sequence. The arrows symbolize activation and T headed lines repression. The numbers in black boxes representing the count of identified genes in the current study. PHYA Phytrochrome A, VRN Vernalization, FLC Flowering Locus C, FT Flowering Locus T, FPA Flowering time control protein, ABA Abscisic acid, ICE inducer of CBF expression, CBF C-repeat binding factor, JA Jasmonate, JAZ Jasmonate zim domain, JAM Jasmonate associated MYC2-like, LEA Late embryogenesis-abundant and COR Cold-responsive.

As mentioned above, several genes are involved in the enhancement of FroT in plants. In addition to the *ICE-CBF-COR* pathway and flowering time genes, we identified nine genes of Jasmonates (JA) in the present study, which play a major role in the *ICE-CBF-COR* pathway (Fig. 2) by activating transcription factors. Activated transcription factors bind to the cis-acting element in the promoter of target genes to increase FroT in plants^24^.

## Conclusion

This study dealt with the identification of QTL regions and putative candidate genes associated with FroT in wheat. GWAS resulted in the identification of 23 QTL regions associated with FroT. The identified QTL regions on chromosome 5A and 5B are in accordance with known genomic regions and candidate genes previously described for FroT in wheat. Moreover, the findings reported here, confirm the results of the previous study of Babben et al.^25^ in regard to polymorphisms in candidate genes for FroT (*CBF-14)* on chromosome 5A. To the best of our knowledge, eight of the detected QTL regions can be assumed to be novel, as these regions were not described in literature before. Furthermore, within the QTL regions on chromosome 1B, 2A, 2B, 3A, 5A and 5B genes with GO terms associated with cold stress response or FroT were identified. The findings reported here give hints to known and previously unknown genome regions and candidate genes, which are putatively associated with FroT in wheat and therefore mark the starting point for further research. Prospectively, these findings will help to develop diagnostic markers for FroT in wheat and to fine map putative candidate genes. Both will foster the better understanding of FroT in wheat and the improvement of winter hardiness and FroT in bread wheat elite breeding pools.

## Supplementary Information

Supplementary Information Legend.

Supplementary Table S1.

Supplementary ﻿Table S2.

Supplementary ﻿Table S3.

Supplementary ﻿Table S4.

Supplementary ﻿Table S5.

Supplementary ﻿Table S6.

Supplementary ﻿Table S7.

Supplementary Figure S1.

Supplementary Figure S2.

Supplementary Figure S3.

Supplementary Figure S4.

## References

[1] Dvorak Jand Akhunov ED (2005). Tempos of gene locus deletions and duplications and their relationship to recombination rate during diploid and polyploid evolution in the aegilops-triticum alliance. Genetics.

[2] Peng JH, Sun D, Nevo E (2011). Domestication evolution, genetics and genomics in wheat. Mol. Breed..

[3] Faris JD, Tuberosa R, Graner A (2014). Wheat domestication: Key to agricultural revolutions past and future. Genomics of plant genetic resources.

[4] Li GQ (2013). Vernalization requirement duration in winter wheat is controlled by TaVRN-A1 at the protein level. Plant J..

[5] Yan L, Ogihara Y, Takumi S, Handa H (2015). Genetic Mechanisms of Vernalization Requirement Duration in Winter Wheat Cultivars. Advances in Wheat Genetics: From Genome to Field.

[6] Armoniene R, Liatukas Z, Brazauskas G (2013). Evaluation of freezing tolerance of winter wheat (*Triticum aestivum* L.) under controlled conditions and in the field. Zemdirbyste-Agriculture.

[7] Ozturk A, Caglar O, Bulut S (2006). Growth and yield response of facultative wheat to winter sowing, freezing sowing and spring sowing at different seeding rates. J. Agron. Crop Sci..

[8] FAO. Food and Agriculture Organization of the United Nations. http://www.fao.org/3/ca4526en/ca4526en.pdf (2019)

[9] Braun HJ and Sãulescu, N.N., Breeding winter and facultative wheat. Central Asia 9.6. FAOSTAT, 2020 http://www.faoorg/3/y4011e/y4011e0fhtm (2002)

[10] Barlow KM, Christy BP, O’leary GJ, Riffkin PA, Nuttall JG (2015). Simulating the impact of extreme heat and frost events on wheat crop production: A review. Field Crops Res..

[11] Trnka M (2014). Adverse weather conditions for European wheat production will become more frequent with climate change. Nat. Clim. Chang..

[12] Gu L (2008). The 2007 eastern US spring freeze: Increased cold damage in a warming world?. Bioscience.

[13] Wu YF (2014). Frost affects grain yield components in winter wheat. N. Z. J. Crop. Hort. Sci..

[14] Nuttall JG (2019). Frost response in wheat and early detection using proximal sensors. J. Agron. Crop Sci..

[15] Vagujfalvi A, Galiba G, Cattivelli L, Dubcovsky J (2003). The cold-regulated transcriptional activator Cbf3 is linked to the frost-tolerance locus Fr-A2 on wheat chromosome 5A. Mol. Genet. Genomics.

[16] Visioni A (2013). Genome-wide association mapping of frost tolerance in barley (*Hordeum vulgare* L.). BMC Genomics.

[17] Liu, W. X. et al. Genetic architecture of winter hardiness and frost tolerance in triticale. *Plos One* 9 (2014) 10.1371/journal.pone.0099848 PMC4057402 24927281

[18] Kaplan F (2007). Transcript and metabolite profiling during cold acclimation of Arabidopsis reveals an intricate relationship of cold-regulated gene expression with modifications in metabolite content. Plant J..

[19] Ding YL, Shi YT, Yang SH (2019). Advances and challenges in uncovering cold tolerance regulatory mechanisms in plants. New Phytol..

[20] Todorovska EG (2014). The expression of CBF genes at Fr-2 locus is associated with the level of frost tolerance in Bulgarian winter wheat cultivars. Biotechnol. Biotechnol. Equip..

[21] Guo J, Ren YK, Tang ZH, Shi WP, Zhou MX (2019). Characterization and expression profiling of the ICE-CBF-COR genes in wheat. PeerJ.

[22] Jin YN (2018). Identification of genes from the ICE-CBF-COR pathway under cold stress in Aegilops-Triticum composite group and the evolution analysis with those from Triticeae. Physiol. Mol. Biol. Plants.

[23] Shi YT, Ding YL, Yang SH (2015). Cold signal transduction and its interplay with phytohormones during cold acclimation. Plant Cell Physiol..

[24] Ritonga FN, Chen S (2020). Physiological and molecular mechanism involved in cold stress tolerance in plants. Plants.

[25] Babben S (2018). Association genetics studies on frost tolerance in wheat (*Triticum aestivum* L.) reveal new highly conserved amino acid substitutions in CBF-A3, CBF-A15, VRN3 and PPD1 genes. BMC Genomics.

[26] Galiba G, Vágújfalvi A, Li C, Soltész A, Dubcovsky J (2009). Regulatory genes involved in the determination of frost tolerance in temperate cereals. Plant Sci..

[27] Dhillon T (2010). Regulation of freezing tolerance and flowering in temperate cereals: The VRN-1 connection. Plant Physiol.

[28] Varshney, R.K. et al. Designing future crops: Genomics-assisted breeding comes of age. Trends Plant Sci. (2021) 10.1016/j.tplants.2021.03.010 33893045

[29] Li X (2021). An integrated framework reinstating the environmental dimension for GWAS and genomic selection in crops. Mol. Plant.

[30] Chen H (2017). A genome-wide association study identifies genetic variants associated with mathematics ability (vol 7, 2017). Sci. Rep..

[31] Korte A, Farlow A (2013). The advantages and limitations of trait analysis with GWAS: a review. Plant Methods.

[32] Wang SC (2014). Characterization of polyploid wheat genomic diversity using a high-density 90 000 single nucleotide polymorphism array. Plant Biotechnol. J..

[33] Allen AM (2017). Characterization of a Wheat Breeders' Array suitable for high-throughput SNP genotyping of global accessions of hexaploid bread wheat (*Triticum aestivum*). Plant Biotechnol. J..

[34] Liu, J. et al. A genome-wide association study of wheat spike related traits in China. *Front. Plant Sci.* 9 (2018) 10.3389/fpls.2018.01584 PMC6220075 30429867

[35] Turuspekov, Y. et al. GWAS for plant growth stages and yield components in spring wheat (*Triticum aestivum* L.) harvested in three regions of Kazakhstan. *BMC Plant Biol.* 17 (2017) 10.1186/s12870-017-1131-2 PMC5688510 29143598

[36] Valluru R, Reynolds MP, Davies WJ, Sukumaran S (2017). Phenotypic and genome-wide association analysis of spike ethylene in diverse wheat genotypes under heat stress. New Phytol..

[37] Sukumaran S, Dreisigacker S, Lopes M, Chavez P, Reynolds MP (2015). Genome-wide association study for grain yield and related traits in an elite spring wheat population grown in temperate irrigated environments. Theor. Appl. Genet..

[38] Zanke C (2014). Genetic architecture of main effect QTL for heading date in European winter wheat. Front. Plant Sci..

[39] Arora, et al. Genome-wide association study of grain architecture in wild wheat *Aegilops tauschii*. *Front. Plant Sci.* 8 (2017) 10.3389/fpls.2017.00886 PMC5450224 28620398

[40] Naruoka Y, Garland-Campbell KA, Carter AH (2015). Genome-wide association mapping for stripe rust (*Puccinia striiformis* F. sp tritici) in US Pacific Northwest winter wheat (*Triticum aestivum* L.). Theor. Appl. Genet..

[41] Zhao, Y., et al. Genome-wide association study reveals the genetic basis of cold tolerance in wheat. *Mol. Breed.* 40 (2020)

[42] Båga M (2007). Identification of quantitative trait loci and associated candidate genes for low-temperature tolerance in cold-hardy winter wheat. Funct. Integr. Genomics.

[43] Zhao Y (2013). Dissecting the genetic architecture of frost tolerance in Central European winter wheat. J. Exp. Bot..

[44] Case AJ, Skinner DZ, Garland-Campbell KA, Carter AH (2014). Freezing tolerance-associated quantitative trait loci in the brundage x coda wheat recombinant inbred line population. Crop Sci..

[45] Fowler, D. B., N'Diaye, A., Laudencia-Chingcuanco, D., & Pozniak, C. J. Quantitative trait loci associated with phenological development, low-temperature tolerance, grain quality, and agronomic characters in wheat (*Triticum aestivum* L.). *Plos One* 11 (2016) 10.1371/journal.pone.0152185 PMC4809511 27019468

[46] Kruse EB (2017). Genomic regions associated with tolerance to freezing stress and snow mold in winter wheat. G3-Genes Genomes Genetics.

[47] Würschum T, Longin CF, Hahn V, Tucker MR, Leiser WL (2017). Copy number variations of CBF genes at the Fr-A2 locus are essential components of winter hardiness in wheat. Plant J..

[48] SASInstitute. SAS Software. Version 9.4. Cary, NC (2019)

[49] IWGSC (2018) Shifting the limits in wheat research and breeding using a fully annotated reference genome. Science 361 (6403) 10.1126/science.aar7191 30115783

[50] Browning SR, Browning BL (2007). Rapid and accurate haplotype phasing and missing-data inference for whole-genome association studies by use of localized haplotype clustering. Am. J. Hum. Genet..

[51] Shin JH, Blay S, McNeney B, Graham J (2006). LDheatmap: An R function for graphical display of pairwise linkage disequilibria between single nucleotide polymorphisms. J. Stat. Softw..

[52] Warnes, G., Gorjanc, G., Leisch, F., & Man, M. Genetics: Population genetics. R package version 1.3.8.1. http://CRAN.R-project.org/package=genetics (2013)

[53] Voss-Fels, K. et al. Subgenomic diversity patterns caused by directional selection in bread wheat gene pools. *Plant Genome* 8 (2015) 10.3835/plantgenome2015.03.0013 33228295

[54] Sannemann, W., Huang, B.E., Mathew, B., Leon, J. Multi-parent advanced generation inter-cross in barley: High-resolution quantitative trait locus mapping for flowering time as a proof of concept. *Mol. Breed*. 35 (2015)

[55] Reif JC, Melchinger AE, Frisch M (2005). Genetical and mathematical properties of similarity and dissimilarity coefficients applied in plant breeding and seed bank management. Crop Sci..

[56] Pritchard JK, Stephens M, Donnelly P (2000). Inference of population structure using multilocus genotype data. Genetics.

[57] Perrier, X., & Jacquemoud-Collet, J.P. DARwin Software. Dissimilarity Analysis and Represntation for Windows. http://darwin.cirad.fr/ (2006)

[58] Earl DA (2012). STRUCTURE HARVESTER: A website and program for visualizing STRUCTURE output and implementing the Evanno method. Conserv. Genet. Resour..

[59] Bradbury PJ (2007). TASSEL: Software for association mapping of complex traits in diverse samples. Bioinformatics.

[60] Spataro G (2011). Genetic diversity and structure of a worldwide collection of *Phaseolus coccineus* L. Theor. Appl. Genet..

[61] Sofalian, O., Mohammadi, S. A., Aharizad, S., Moghaddam, M., & Shakiba, M.R. Mapping of QTLs for frost tolerance and heading time using SSR markers in bread wheat. *Afr. J. Biotechnol*. 8(20) (2009)

[62] Motomura Y, Kobayashi F, Iehisa JC, Takumi S (2013). A major quantitative trait locus for cold-responsive gene expression is linked to frost-resistance gene Fr-A2 in common wheat. Breed. Sci..

[63] Guerra D et al. (2021) Extensive allele mining discovers novel genetic diversity in the loci controlling frost tolerance in barley. Theoretical and Applied Genetics 1–17 10.1007/s00122-021-03985-x PMC8866391 34757472

[64] Royo C, Nazco R, Villegas D (2014). The climate of the zone of origin of Mediterranean durum wheat (*Triticum durum* Desf.) landraces affects their agronomic performance. Genet. Resour. Crop Evol..

[65] Venske E, Dos Santos RS, Busanello C, Gustafson P, de Oliveira AC (2019). Bread wheat: A role model for plant domestication and breeding. Hereditas.

[66] Kajla M (2015). Increase in wheat production through management of abiotic stresses: a review. J. Appl. Nat. Sci..

[67] Muthamilarasan M, Prasad M (2014). An overview of wheat genome sequencing and its implications for crop improvement. J. Genet..

[68] Li J, Yang J, Li Y, Ma L (2020). Current strategies and advances in wheat biology. Crop J..

[69] Chen Y (2019). Application of image-based phenotyping tools to identify QTL for in-field winter survival of winter wheat (*Triticum aestivum* L.). Theor. Appl. Genetics.

[70] Sieber AN, Longin CF, Leiser WL, Würschum T (2016). Copy number variation of CBF-A14 at the Fr-A2 locus determines frost tolerance in winter durum wheat. Theor. Appl. Genet..

[71] Franklin KA, Whitelam GC (2007). Light-quality regulation of freezing tolerance in Arabidopsis thaliana. Nat. Genet..

[72] Wang F (2020). Crosstalk of PIF4 and DELLA modulates CBF transcript and hormone homeostasis in cold response in tomato. Plant Biotechnol. J..

[73] Novák A (2016). Light-quality and temperature-dependent CBF14 gene expression modulates freezing tolerance in cereals. J. Exp. Bot..

[74] Gierczik K (2017). Circadian and light regulated expression of CBFs and their upstream signalling genes in barley. Int J Mol Sci.

[75] Takumi S (2003). Cold-specific and light-stimulated expression of a wheat (*Triticum aestivum* L.) Cor gene Wcor15 encoding a chloroplast-targeted protein. J. Exp. Bot..

[76] Limin AE, Fowler DB (2006). Low-temperature tolerance and genetic potential in wheat (*Triticum aestivum* L.): Response to photoperiod, vernalization, and plant development. Planta.

[77] Huang XS, Wang W, Zhang Q, Liu JH (2013). A basic helix-loop-helix transcription factor, PtrbHLH, of *Poncirus trifoliata* confers cold tolerance and modulates peroxidase-mediated scavenging of hydrogen peroxide. Plant Physiol..

[78] Jiang Y, Yang B, Deyholos MK (2009). Functional characterization of the Arabidopsis bHLH92 transcriptionfactor in abiotic stress. Mol Genet Genomics.

[79] Kiribuchi K (2005). Involvement of the basic helix-loop-helix transcription factor RERJ1 in wounding and drought stress responses in rice plants. Biosci. Biotechnol. Biochem..

[80] Ogo Y (2006). Isolation and characterization of IRO2, a novel iron-regulated bHLH transcription factor in graminaceous plants. J. Exp. Bot..

[81] Wang YJ (2003). A rice transcription factor OsbHLH1 is involved in cold stress response. Theor. Appl. Genet..

[82] Xu W (2014). The grapevine basic helix-loop-helix (bHLH) transcription factor positively modulates CBF-pathway and confers tolerance to cold-stress in Arabidopsis. Mol. Biol. Rep..

[83] Wang L, Xiang L, Hong J, Xie Z, Li B (2019). Genome-wide analysis of bHLH transcription factor family reveals their involvement in biotic and abiotic stress responses in wheat (*Triticum aestivum* L.). 3 Biotech..

[84] Goossens J, Mertens J, Goossens A (2017). Role and functioning of bHLH transcription factors in jasmonate signalling. J. Exp. Bot..

[85] Sasaki-Sekimoto Y (2013). Basic helix-loop-helix transcription factors JASMONATE-ASSOCIATED MYC2-LIKE1 (JAM1), JAM2, and JAM3 are negative regulators of jasmonate responses in Arabidopsis. Plant Physiol..

[86] Xiang L (2019). The cold-induced transcription factor bHLH112 promotes artemisinin biosynthesis indirectly via ERF1 in Artemisia annua. J. Exp. Bot..

[87] Jiang L (2019). The AabHLH35 transcription factor identified from Anthurium andraeanum is involved in cold and drought tolerance. Plants.

[88] Yamasaki K (2004). Solution structure of the B3 DNA binding domain of the Arabidopsis cold-responsive transcription factor RAV1. Plant Cell.

[89] Chen L (2016). Expansion and stress responses of AP2/EREBP superfamily in Brachypodium distachyon. Sci. Rep..

[90] Chen HC, Cheng WH, Hong CY, Chang YS, Chang MC (2018). The transcription factor OsbHLH035 mediates seed germination and enables seedling recovery from salt stress through ABA-dependent and ABA-independent pathways, respectively. Rice.

[91] Dong Y (2014). A novel bHLH transcription factor PebHLH35 from Populus euphratica confers drought tolerance through regulating stomatal development, photosynthesis and growth in Arabidopsis. Biochem. Biophys. Res. Commun..

