# Supplementary material for: Genome wide association study of frost tolerance in wheat
Source: Sci Rep. 2022 Mar 28;12:5275. doi: 10.1038/s41598-022-08706-y (PMC8960795; doi:10.1038/s41598-022-08706-y)
Supplement: Supplementary file 1 — Supplementary Information Legend. [file 41598_2022_8706_MOESM1_ESM.docx]

**Supplemental Material:**

Supplementary Table S1: 276 genotypes used in this study with information about source, origin and improvement level.

Supplementary Table S2: Summary of high and low confidential (HC and LC) genes located within the QTL regions significantly associated (LOD≥4) with frost tolerance (FroT).

Supplementary Table S3: List of calculated LD decay for each chromosome, separately.

Supplementary Table S4: Summary of 53 SNP markers significantly associated (LOD≥4) with FroT.

Supplementary Table S5: List of high and low confidential (HC and LC) genes located within the identified QTL regions and GO terms associated with frost or cold tolerance.

Supplementary Table S6: List of candidate genes identified via BLASTn.

Supplementary Table S7: Comparison of identified QTLs in the present study with reported QTL in previous studies.

Figure S1: DeltaK plot: Number of optimal detected clusters (K= 3) according Bayesian clustering approach.

Figure S2: Principal coordinate analysis (PCoA) according to Structure grouping of 276 wheat genotypes. Legend: blue dots: genotypes assigned to Structure group 1, orange dots: genotypes assigned to Structure group 2, gray dots: genotypes assigned to structure group 3, yellow dots: genotypes assigned to the admixed group.

Figure S3: Quantile-Quantile (QQ) plot of the comparison of the different applied methods for GWAS: QQ plot indicates performance of the mixed linear model and general mixed model for GWAS.

Figure S4: Manhattan plot: GWAS results for FroT of wheat based on 276 genotypes and 17,566 SNP markers.
