# Supplementary figures and images for: Genome wide association study of frost tolerance in wheat
Source: Sci Rep. 2022 Mar 28;12:5275. doi: 10.1038/s41598-022-08706-y (PMC8960795; doi:10.1038/s41598-022-08706-y)

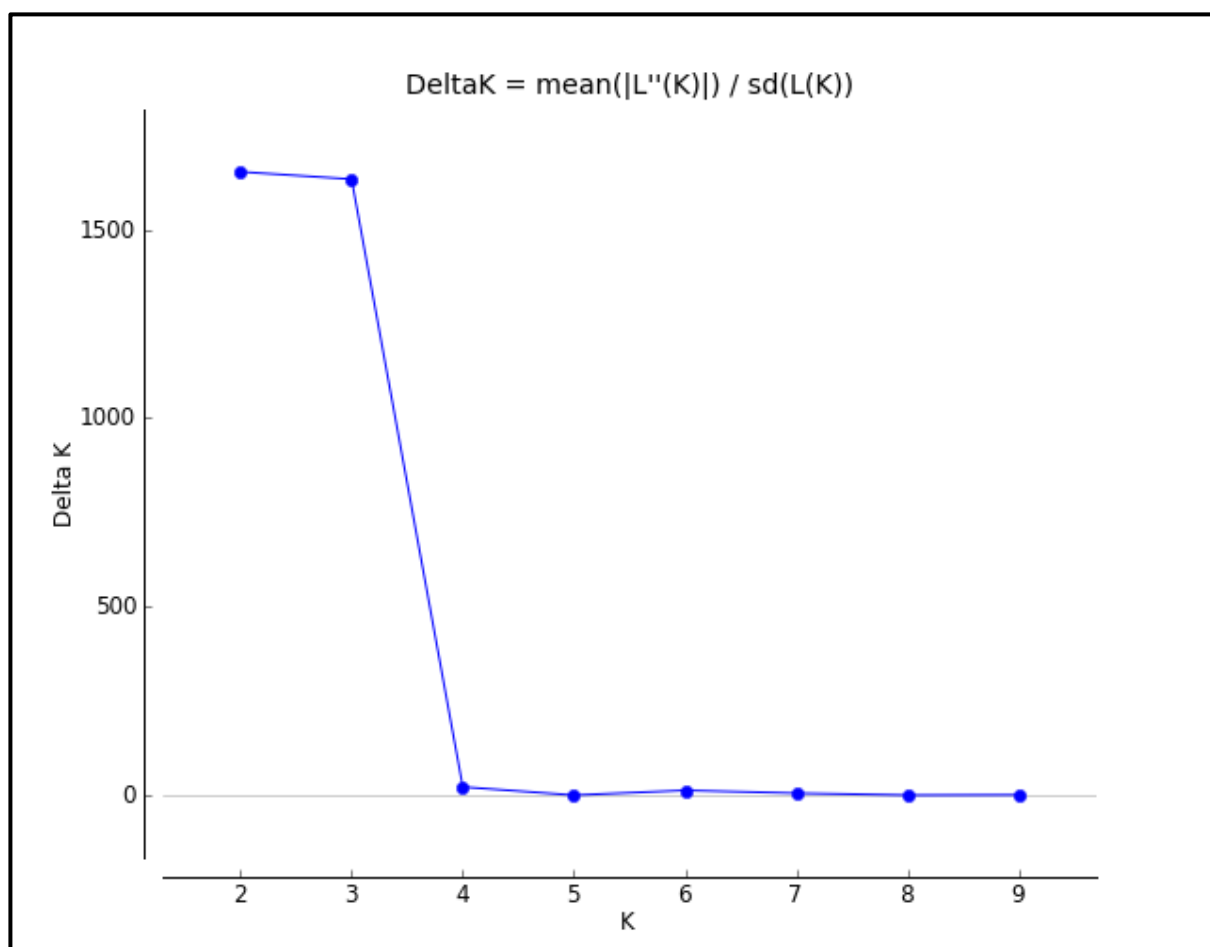

Supplement: Supplementary file 9 — Supplementary Figure S1. [file 41598_2022_8706_MOESM9_ESM.pdf]

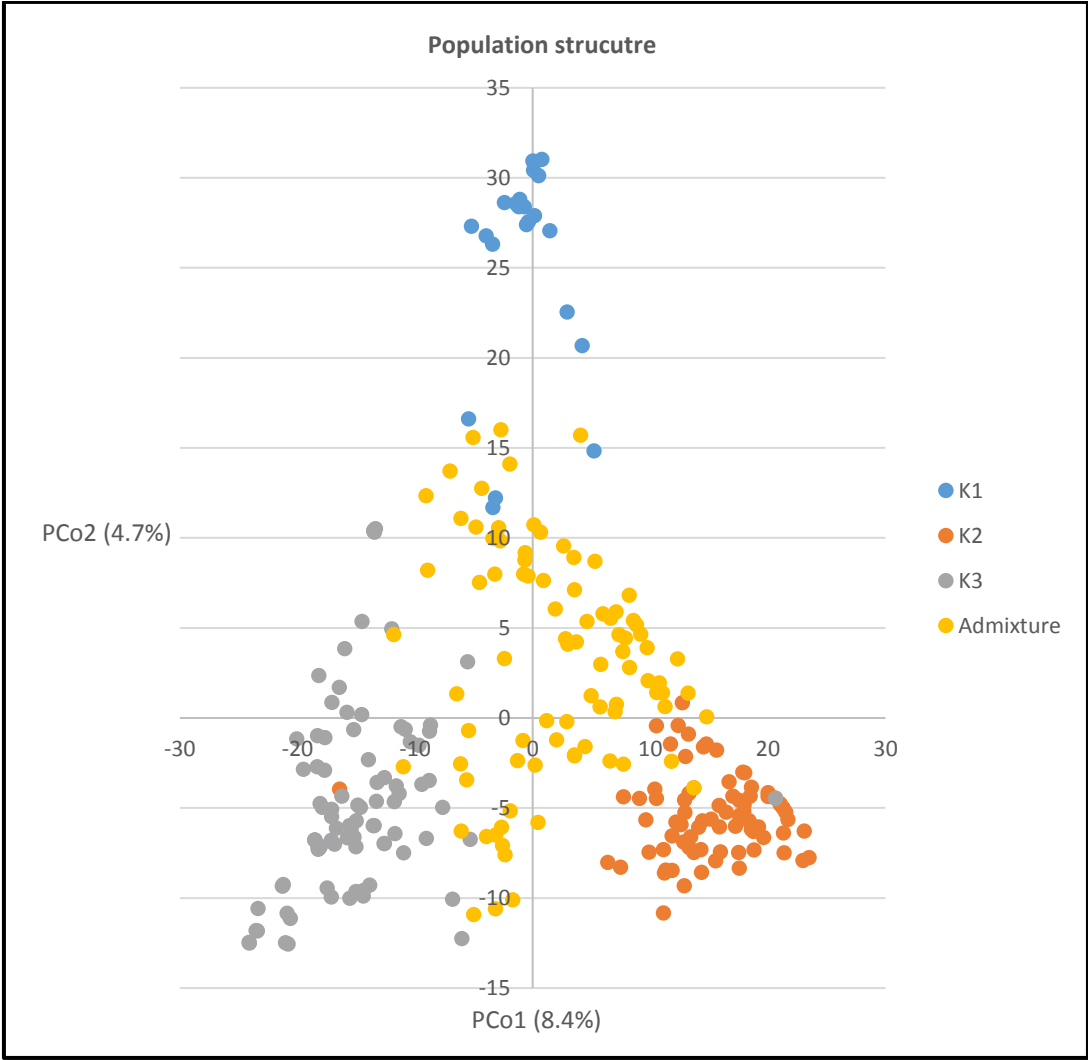

Supplement: Supplementary file 10 — Supplementary Figure S2. [file 41598_2022_8706_MOESM10_ESM.pdf]

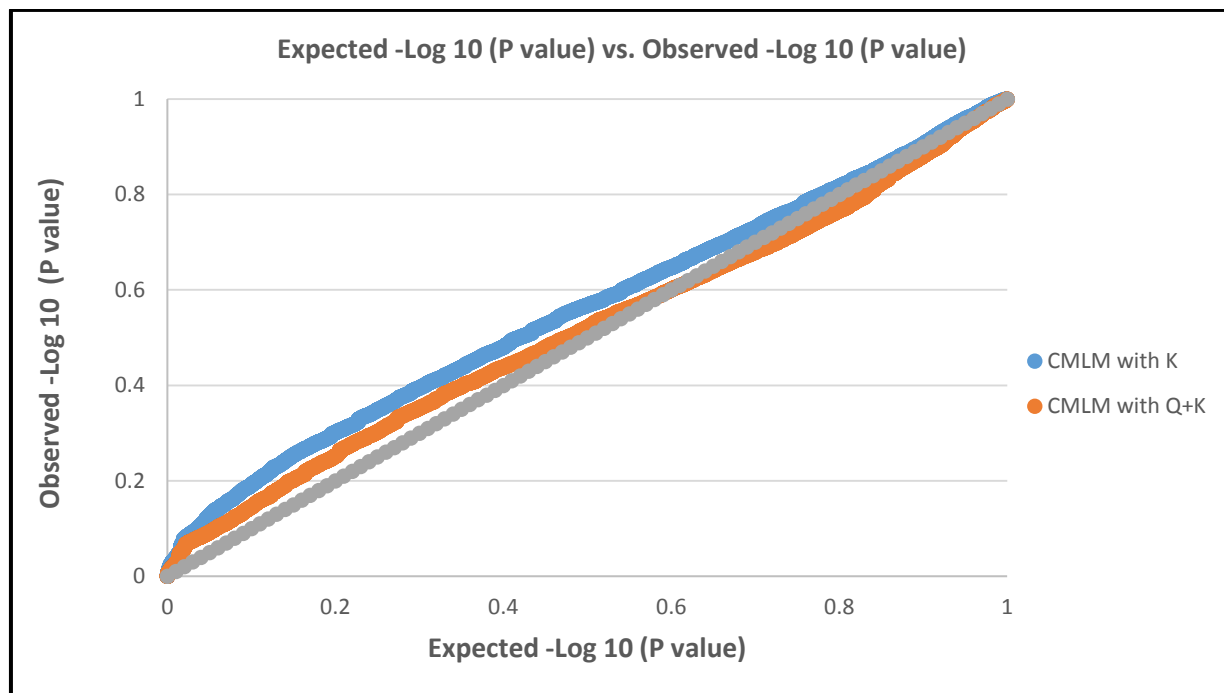

Supplement: Supplementary file 11 — Supplementary Figure S3. [file 41598_2022_8706_MOESM11_ESM.pdf]

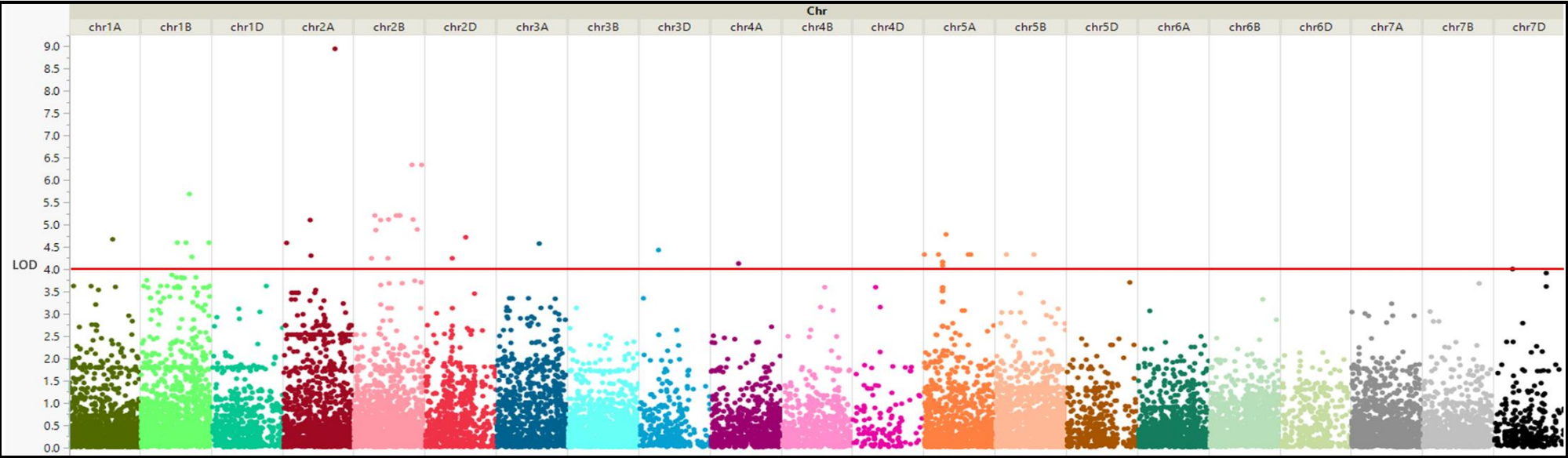

Supplement: Supplementary file 12 — Supplementary Figure S4. [file 41598_2022_8706_MOESM12_ESM.pdf]
